# Custo-Efetividade do Emprego do Escore de Cálcio Coronariano na Orientação para Terapia na Prevenção Primária, na População Brasileira

**DOI:** 10.36660/abc.20220293

**Published:** 2022-06-06

**Authors:** Ilan Gottlieb

**Affiliations:** 1 Casa de Saúde São José Rio de Janeiro RJ Brasil Casa de Saúde São José, Rio de Janeiro, RJ – Brasil

**Keywords:** Doenças Cardiovasculares/prevenção e controle, Infarto do Miocárdio, Acidente Vascular Cerebral, Doença Arterial Coronariana, Aterosclerose, Fatores de Risco, Placa Aterosclerótica, Estatinas, Análise de Custo-Benefício

As estatísticas de doenças cardiovasculares (DCV) nunca deixam de impressionar até mesmo o médico mais experiente. Um terço das mortes no mundo ainda se deve a causas cardiovasculares (85% delas são infarto do miocárdio e acidente vascular cerebral), e 75% ocorrem em países de renda média a baixa.^1,2^ Metade das pessoas que morreram de infarto do miocárdio nunca apresentou sintomas antes do trágico evento, e a maioria nunca teve o diagnóstico de doença arterial coronariana.^3^ Apesar de nossos melhores esforços, a prevalência da doença isquêmica do coração (DIC) vem aumentando constantemente nos últimos 30 anos em todo o mundo devido ao envelhecimento populacional, mas mesmo padronizando por idade, a prevalência tem se mantido estável e não caiu.^4^

A calcificação coronária é quase sempre um marcador de aterosclerose. O escore de cálcio coronariano (ECC) é um número que quantifica a calcificação coronariana como um substituto para a carga aterosclerótica coronariana total. Embora a calcificação resulte da cicatrização da placa, as placas de maior risco tendem a ter componentes não calcificados proporcionalmente maiores;^5^ a ECC provou ser um forte preditor de eventos de DCV e DIC em vários grandes e sólidos estudos de base populacional.^6^

A prevenção primária é orientada e titulada pelo risco de DCV, ou seja, pacientes com maior risco devem ter tratamento de maior intensidade, e pacientes de baixo risco podem não necessitar de tratamento além do aconselhamento geral de saúde. A ECC determina o risco cardiovascular melhor do que a avaliação clínica e as calculadoras de risco clínico porque a DCV tem uma fisiopatologia tão diversa e complexa, com tantos fatores de risco diferentes, que compilar todos os fatores de risco em uma calculadora é ineficaz e impreciso. Além disso, os fatores de risco são tão comuns que não conseguem diferenciar quem terá um evento e quem não terá. Por exemplo, a prevalência de 1 fator de risco principal (além da idade) é muito alta entre pessoas de 40 anos que desenvolvem DIC,^7^ mas também é muito alta entre aquelas que não desenvolvem DIC.^8^ Em vez de focar em como adivinhar quem tem DCV, devemos nos concentrar no diagnóstico precoce de DCV pré-clínica, e o escore de cálcio coronariano é provavelmente a melhor ferramenta disponível, pois é preciso, relativamente barato, amplamente disponível e custo-efetivo em múltiplos cenários clínicos e populações.^9^

Os ABC deste mês trazem um artigo muito importante que investiga o custo-efetividade da ECC no Brasil.^10^ Como o escâner, os medicamentos e outros custos de saúde variam em todo o mundo, é importante realizar análises de custo-efetividade localmente para orientar melhor as políticas nacionais de saúde. Os autores demonstraram que, entre os pacientes clinicamente classificados como de risco intermediário, que seriam recomendados ou considerados para tratamento com estatinas de intensidade moderada pelas diretrizes clínicas atuais, a introdução do ECC é custo-efetiva em todos os cenários analisados. Não apenas um aumento na intensidade das estatinas seria recomendado para a população de pacientes com ECC > 100 (25% da coorte) que, de outra forma, estaria recebendo apenas tratamento de intensidade moderada, mas talvez mais importante seja o fato de que aproximadamente 45% da população de pacientes seria retirada da terapia médica por ter ECC=0. O custo do ECC é compensado pela redução das taxas de eventos em ECC>100 e pela economia da suspensão de estatinas a longo prazo entre aqueles com ECC=0.

Alguns recursos importantes estão faltando na análise, uma vez que não mostraram como coletaram os dados de custo e não forneceram análise de sensibilidade. No entanto, apesar dessas deficiências, seu trabalho é valioso para o planejamento da saúde da população no Brasil. Juntamente com outros dados de custo-efetividade que analisaram tecnologias semelhantes,^11^ seu artigo reforça o escore de cálcio como uma ferramenta valiosa para orientar e titular a terapia médica e melhorar a adesão do paciente às mudanças comportamentais necessárias
