# Supplementary material for: Custo-Efetividade do Emprego do Escore de Cálcio Coronariano na Orientação para Terapia na Prevenção Primária, na População Brasileira
Source: Arq Bras Cardiol. 2022 Jun 6;118(6):1132–3. [Article in Portuguese] doi: 10.36660/abc.20220293 (PMC9345148; doi:10.36660/abc.20220293)
Supplement: Translation

# Translation: Cost-Effectiveness of Using the Coronary Calcium Score to Guide Therapeutic Decisions in Primary Prevention in the Brazilian Population

**Authors:** Ilan Gottlieb

**Affiliations:** 1 Rio de Janeiro RJ Brazil Casa de Saúde São José, Rio de Janeiro, RJ – Brazil

**Keywords:** Cardiovascular Diseases/prevention and control, Myocardial Infarction, Stroke, Coronary Artery Disease, Atherosclerosis, Risk Factors, Plaque Atherosclerotic, Statins, Cost-Benefit Analysis

Cardiovascular diseases (CVD) statistics never fail to impress even the most hardened and experienced physician. One-third of deaths worldwide are still due to cardiovascular causes (85% of those are myocardial infarction and stroke), and 75% occur in mid-to-low income countries.^1,2^ Half the people who died of myocardial infarction never had symptoms before the tragic event, and most never had the diagnosis of coronary artery disease.^3^ Despite our best efforts, the prevalence of ischemic heart disease (IHD) has been steadily rising for the last 30 years worldwide due to population aging, but even if we standardize by age, the prevalence has been stable, and notwithstanding fallen. This fact highlights the importance of lifestyle changes that allow for a healthier heart and the need to focus on implementing existing cost-effective policies and interventions.^4^

Coronary calcification is almost always a marker of atherosclerosis. The coronary calcium score (CCS) is a number that quantifies coronary calcification as a surrogate for total coronary atherosclerotic burden. Even though calcification results from plaque healing, higher-risk plaques tend to have proportionately greater non-calcified components;^5^ CCS has been proven to be a strong predictor of CVD and IHD events in multiple large, solid, population-based studies.^6^

Primary prevention is guided and titrated by CVD risk, i.e., patients with higher risk should have higher intensity treatment, and low-risk patients may require no treatment besides general healthcare counseling. CCS determines cardiovascular risk better than clinical assessment and clinical risk calculators because CVD has such diverse and complex pathophysiology, with so many different risk factors, that compiling all risk factors in a calculator is ineffective and inaccurate. Additionally, risk factors are so common that they fail to differentiate who will have an event and who will not. For instance, the prevalence of 1 major risk factor (aside from age) is very high among persons aged 40 years who develop IHD,^7^ but it is also very high among those who do not develop IHD.^8^ Instead of focusing on how to guess who has CVD, we should focus on the early diagnosis of preclinical CVD, and coronary calcium score is probably the best tool available, for it is accurate, relatively cheap, widely available, and cost-effective in multiple clinical scenarios and populations.^9^

This month's ABC brings a very important article that investigates the cost-effectiveness of CCS in Brazil.^10^ Since scanning, medications and other healthcare costs vary worldwide, it is important to perform cost-effectiveness analysis locally to guide national healthcare policies better. The authors demonstrated that, among patients clinically classified as intermediate risk, who would be recommended or considered for moderate intensity statin treatment by current clinical guidelines, the introduction of CCS is cost-effective in all analyzed scenarios. Not only an increase in statin intensity would be recommended for the patient population with CCS>100 (25% of the cohort) who would otherwise be taking only moderate-intensity treatment, but perhaps more important is the fact that approximately 45% of the patient population would be withdrawn from medical therapy since they have CCS=0. The cost of the CCS scan is compensated by lowering event rates in CCS>100 and the savings from long-term statin suspension among those with CCS=0.

Some important features are missing from the analysis, since they did not show how they collected cost data and did not provide sensitivity analysis. Nevertheless, despite these shortcomings, their paper is valuable for population healthcare planning in Brazil. Together with other cost-effectiveness data that analyzed similar technologies,^11^ their paper reinforces calcium score as a valuable tool to guide and titrate medical therapy and improve patient adherence to necessary behavioral changes.
